# Association Between Dietary Inflammatory Index and Heart Failure: Results From NHANES (1999–2018)

**DOI:** 10.3389/fcvm.2021.702489

**Published:** 2021-07-06

**Authors:** Zuheng Liu, Haiyue Liu, Qinsheng Deng, Changqing Sun, Wangwei He, Wuyang Zheng, Rong Tang, Weihua Li, Qiang Xie

**Affiliations:** ^1^Department of Cardiology, The First Affiliated Hospital of Xiamen University, Xiamen, China; ^2^Department of Laboratory Medicine, The First Affiliated Hospital of Xiamen University, Xiamen, China; ^3^Xiamen Key Laboratory of Genetic Testing, Xiamen, China

**Keywords:** heart failure, dietary inflammatory index, nutrition, national health and nutrition examination survey, cardiovascular and cerebrovascular diseases

## Abstract

**Objective:** To explore the relationship between dietary inflammatory index (DII) and heart failure (HF) in participants with cardiovascular and cerebrovascular diseases.

**Methods:** NHANES (1998–2018) data were collected and used to assess the association of HF with DII. Twenty-four-hour dietary consumptions were used to calculate the scores of DII. Demographic characteristics and physical and laboratory examinations were collected for the comparison between HF and non-HF groups. Logistic regression analysis and random forest analysis were performed to calculate the odds rate and determine the potential beneficial dietary components in HF.

**Results:** A total of 19,067 cardiac-cerebral vascular disease participants were categorized as HF (*n* = 1,382; 7.25%) and non-HF (*n* = 17,685; 92.75%) groups. Heart failure participants had higher levels of DII score compared with those in the non-HF group (0.239 ± 1.702 vs. −0.145 ± 1.704, *p* < 0.001). Compared with individuals with T1 (DII: −3.884 to −0.570) of DII, those in T3 (DII: 1.019 to 4.598) had a higher level of total cholesterol (4.49 ± 1.16 vs. 4.75 ± 1.28 mmol/L, *p* < 0.01), globulin (29.92 ± 5.37 vs. 31.29 ± 5.84 g/L, *p* < 0.001), and pulse rate (69.90 ± 12.22 vs. 72.22 ± 12.77, *p* < 0.001) and lower levels of albumin (40.76 ± 3.52 vs. 39.86 ± 3.83 g/L, *p* < 0.001), hemoglobin (13.76 ± 1.65 vs. 13.46 ± 1.77 g/dl, *p* < 0.05), and hematocrit (40.83 ± 4.69 vs. 40.17 ± 5.01%, *p* < 0.05). The odds rates of HF for DII from the logistic regression were 1.140, 1.158, and 1.110 in models 1, 2, and 3, respectively. In addition, from the results of random forest analysis, dietary magnesium, fiber, and beta carotene may be essential in HF.

**Conclusion:** Dietary inflammatory index was positively associated with HF in US adults, and dietary intervention might be a promising method in the therapy of HF.

## Introduction

Chronic heart failure (HF) is a complicated syndrome that occurs with a high probability at the end stage of various cardiovascular diseases (CVDs). Intestinal congestion is a common feature of HF, which is always contributed to anorexia or poor appetite (1). The variety of dietary supplement pattern had been reported to be linked with the progression of HF. A Western dietary pattern with a higher intake of high-fat products is closely associated with the risk of HF (2). On the contrary, a Mediterranean dietary pattern, which contains high consumptions of vegetables or fish, reduces the risk of HF (3).

The connection between dietary consumption and inflammation has been proposed for many years (4). Although sufficient energy or nutrients supply potentially postpones the evolution of cardiac cachexia (5), the diet-related inflammation should not be neglected. The dietary inflammation index (DII), a widely used scoring system in evaluating the levels of inflammation derived from nutrient supplements, was developed by Cavicchia et al. (6) and updated by Shivappa et al. (7).

An inappropriate dietary pattern is closely related with higher inflammatory factors. For example, a Western diet increases the level of CRP and IL-6 (8), while a Mediterranean diet is associated with lower inflammation factors (9).

Previous studies suggested that IL-1, IL-6, TNF-α, and IFN-γ are increased in HF patients (10). Inflammatory factors lead to anorexia and promote protein degradation in skeletal muscles (11). In addition, inflammatory factors increase cardiac apoptosis and impair cardiac function (12). Importantly, evidence from a clinical trial (CANTOS) indicated that the application of IL-1β receptor inhibitor reduced cardiovascular events (13). In addition, anakinra exhibited favorable trends in reducing high-sensitivity CRP and NT-proBNP in HF with a preserved ejection fraction (14).

Since HF is regarded as an inflammatory-related disease (15, 16) and closely linked with nutrient intake, the assessment of diet-related inflammation would be essential. In order to explore the underlying beneficial nutrients and provide some clues in the therapeutics of HF, we investigated the connections between DII score and HF from the NHANES data.

## Materials and Methods

### Study Population and Design

Data from NHANES 1999–2018 were combined to increase the sample sizes. In order to alleviate the effects of confounding factors, participants with a diagnostic history of coronary artery disease, prediabetes, diabetes, hypertension, heart attack, stroke, or angina were regarded as the control group, while participants who were accompanied with at least one of those basic diseases and HF was defined as the HF group. The verification of HF is based on the questionnaire from MCQ by asking “Someone ever told you had congestive heart failure?” The demographic information and characteristics, which consist of the age, gender, BMI, race, education, income, and current smoking status, were obtained by interviewing. The present study is based on a secondary date analysis which lacked personal identifiers and does not need institutional reviewing.

### Calculation of the Dietary Inflammation Index

The DII is a scoring system in evaluating the potential inflammatory levels of dietary components, which was developed by Shivappa through literature review (7). DII calculates the inflammation effects of dietary consumption from 45 nutrients. The calculation of DII is based on the addition of each component's score from the diet consumed in 24 h, including the score from the pro-inflammatory and anti-inflammatory diet. Briefly, the Z score is a value obtained by subtracting the Global daily mean intake and divided by the standard deviation, and then, the value is converted to a percentile score, followed by doubling each percentile score and subtracting “1” to achieve a symmetrical distribution. Then, the percentile value is multiplied by the corresponding “overall inflammation effect score.” By summing each DII score, we can achieve an individual “overall DII score.” In this study, 26 nutrients were used for the calculation of the DII score, which includes alcohol, vitamin B12/B6, β-carotene, caffeine, carbohydrate, cholesterol, energy, total fat, fiber, folic acid, Fe, Mg, MUFA, niacin, n-3 fatty acids, protein, PUFA, riboflavin, saturated fat, Se, thiamin, vitamins A/C/E, and Zn. Importantly, even if the nutrients applied for the calculation of DII is <30, the DII scores are still available (7).

### Statistical Analysis

The data were processed by R or SPSS 20.0. Student's *t*-test or one-way ANOVA was performed for the comparison of continuous variables followed by LSD or Dunnett's T3 for *post-hoc* multiple comparisons, while chi-square test was performed for comparing the constituent ratio of each group. Levene statistic was used for the homogeneity of the variance test. Logistic regression was performed to assess the association between HF and DII after adjusting for covariates. Random forest analysis was performed using the dietary and examination data using R and 10-fold cross-validation. Receiver operative characteristic curve (ROC) were plotted and area under curves (AUCs) were compared using the pROC package in R (17). Random forest model constructed by Machine learning was used to calculate the score of AUC and explore the possibly beneficial nutrients in HF. In all analyses, differences were considered statistically significant at a value of *p* < 0.05.

## Results

### Demographic Characteristics of Participants

**Figure 1:**
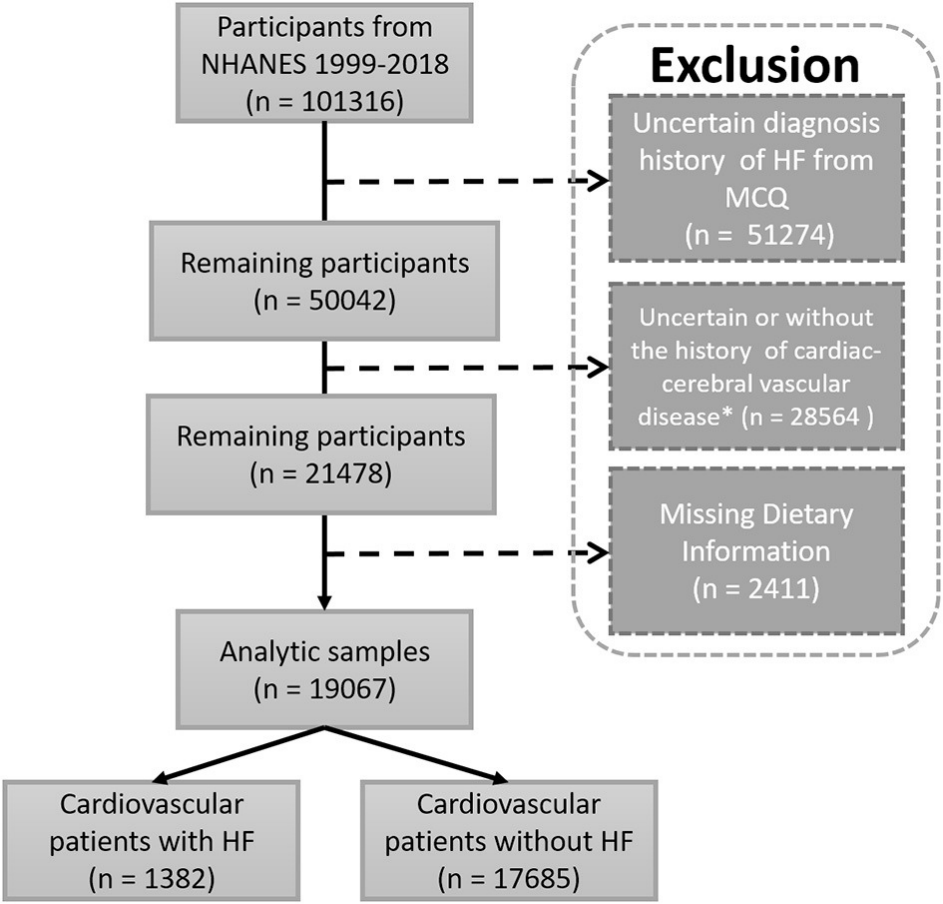
Flow chart of study participants. Sample selection and exclusion criteria for the comparison of HF and non-HF participants, as well as the association between DII and HF. MCQ, the questionnaire of Medical Conditions; NHANES, National Survey of the National Center for Health Statistics; ^*^Including one of the following diagnostic history: hypertension, diabetes, prediabetes, coronary artery disease, heart attack, stroke, angina.

A total of 101,316 participants were included from NHANES 1999–2018. After excluding participants without information of HF diagnosis, uncertain cardiac-cerebral vascular disease, and missing essential dietary information for the calculation of DII, 19,067 participants were obtained for the statistical analysis. Then, they were divided into two groups including the HF (Cardiovascular patients with HF, *n* = 1,382) and non-HF (Cardiovascular patients without HF, *n* = 17,685) groups (Figure 1). The characteristics of analysis samples are shown in Table 1. The prevalence of HF in cardiovascular and cerebrovascular diseases participants was 7.25%. The enrolled participants included 778 (56.30%) and 8,472 (47.91%) male participants with mean ages of 67.82 ± 12.16 and 58.82 ± 15.55 years old in the HF and non-HF groups, respectively Heart failure participants had a higher BMI (31.89 ± 8.06 vs. 30.74 ± 7.14 kg/m^2^, *p* < 0.001) and waist circumference (108.78 ± 17.01 vs. 104.35 ± 15.71, *p* < 0.001) compared with the non-HF group. In addition, the HF participants had a lower education and annual family income. Moreover, the composition of race is also different between these two groups. It is also not surprising that the prevalence of diabetes, coronary artery disease, angina, heart attack, and stroke was higher in participants in the HF group than that of the non-HF group, as these basic diseases would increase the incidence of HF.

**Table 1:** Demographics and characteristics of participants, from NHANES 1999–2018.

**Characteristic**	**HF** **(*N* = 1,382; 7.25%)**	**Non-HF** **(*N* = 17,685; 92.75%)**	***p-*values**
**Age, years, Mean** **±*****SD***	67.82 ± 12.16	58.82 ± 15.55	<0.001
**Gender**, ***N*** **(%)**			
Male	778 (56.30%)	8,472 (47.91%)	<0.001
Female	604 (43.70%)	9,213 (52.10%)	
**BMI, kg/m**^**2**^, ***N*** **(%)**			
<20	34 (2.46%)	412 (2.33%)	0.001
20–25	195 (14.11%)	2,959 (16.73%)	
25–30	386 (27.93%)	5,720 (32.34%)	
≥30	695 (50.29%)	8,225 (46.51%)	
Mean ±*SD*	31.89 ± 8.06	30.74 ± 7.14	<0.001
Missing	72 (5.21%)	369 (2.09%)	
**Waist, cm, Mean** **±*****SD***	108.78 ± 17.01	104.35 ± 15.71	<0.001
**Race**, ***N*** **(%)**			
Mexican American	130 (9.41%)	2,481 (14.03%)	<0.001
Other Hispanic	90 (6.51%)	1,395 (7.89%)	
Non-Hispanic white	748 (54.12%)	8,003 (45.25%)	
Non-Hispanic black	350 (25.33%)	4,388 (24.81%)	
Other race or multi-racial	64 (4.63%)	1,418 (8.02%)	
**Education**, ***N*** **(%)**			
<High school	530 (38.35%)	4,963 (28.06%)	<0.001
High school	344 (24.89%)	4,234 (23.94%)	
>High school	507 (36.69%)	8,463 (47.85%)	
Missing	1 (0.07%)	25 (0.14%)	
**Annual family income**, ***N*** **(%)**			
<20,000 USD	532 (38.50%)	4,793 (27.10%)	<0.001
≥20,000 USD	789 (57.09%)	12,159 (68.75%)	
Missing	61 (4.41%)	733 (4.14%)	
**Current smoking status**, ***N*** **(%)**			
Smoking	266 (19.25%)	3,290 (18.60%)	<0.001
Non-smoking	588 (42.55%)	5,503 (31.12%)	
Missing	528 (38.21%)	8,892 (50.28%)	
**Hypertension**, ***N*** **(%)**	1,156 (83.80%)	14,414 (81.60%)	0.043
**Diabetes**, ***N*** **(%)**	593 (44.40%)	4,848 (28.30%)	<0.001
**Prediabetes**, ***N*** **(%)**	82 (14.50%)	1,925 (19.40%)	0.004
**Coronary artery disease**, ***N*** **(%)**	580 (43.30%)	1,265 (7.20%)	<0.001
**Angina**, ***N*** **(%)**	360 (26.70%)	876 (5.00%)	<0.001
**Heart attack**, ***N*** **(%)**	651 (47.40%)	1,261 (7.10%)	<0.001
**Stroke**, ***N*** **(%)**	301 (21.90%)	1,381 (7.80%)	<0.001

*Data are presented as N% (χ^2^-test) or Mean ± SD (independent t-test)*.

### Physical and Laboratory Examinations of Participants

The results of examinations are shown in Supplementary Table 1. The DII scores ranged from −3.884 to 4.598 and −4.949 to 4.422 in the HF and non-HF groups, respectively Heart failure participants showed a slight increasing trend of WBC (7.60 ± 2.66 vs. 7.38 ± 4.00, *p* = 0.051) and had a higher DII score (0.239 ± 1.702 vs. −0.145 ± 1.704, *p* < 0.001) when compared with the non-HF group. Although the average BMI and waist were higher in HF participants, they had an interesting blood lipid profile, which had a lower total cholesterol (4.61 ± 1.20 vs. 5.05 ± 1.14 mmol/L, *p* < 0.001), HDL (1.26 ± 0.41 vs. 1.36 ± 0.42 mmol/L, *p* < 0.001), LDL (2.58 ± 0.99 vs. 2.91 ± 0.93 mmol/L, *p* < 0.001), and a slightly higher triglyceride (1.77 ± 1.92 vs. 1.62 ± 1.34 mmol/L, p = 0.055) when compared with non-HF participants. Additionally, HF groups had lower levels of albumin (40.27 ± 3.66 vs. 41.63 ± 3.42 g/L, *p* < 0.001), while there was no significance in ALT, AST, and blood sodium. BUN (7.43 ± 4.32 vs. 5.41 ± 2.45 mmol/L, *p* < 0.001), Cr (1.30 ± 0.96 vs. 0.97 ± 0.58 mg/dl, *p* < 0.001), and UA (386.22 ± 11.56 vs. 339.32 ± 88.29 μmol/L, *p* < 0.001) were increased in HF patients which indicated their impairment of renal function. In addition, the plus rate in HF and non-HF are similar though they showed a significant difference (70.84 ± 12.46 vs. 72.59 ± 12.79, *p* < 0.001). Moreover, the levels of hemoglobin (13.55 ± 1.72 g/dl vs. 13.98 ± 1.55 g/dl, *p* < 0.001) and hematocrit (40.29 ± 4.90 vs. 41.35 ± 4.38, *p* < 0.001) in HF were slightly lower than in the non-HF groups. Another interesting finding is that, there is no significant difference in the systolic blood pressure, but the HF group had a lower diastolic blood pressure (66.48 ± 16.20 mmHg vs. 71.10 ± 14.97 mmHg, *p* < 0.001).

### Characteristics and Examinations of HF Participants by Tertiles of Dietary Inflammatory Index

In order to further investigate the correlation between DII and HF, DII was divided by tertiles (Table 2). The DII score ranges from −3.884 to −0.570, −0.566 to 1.019, and 1.019 to 4.598 in tertile 1 (T1), tertile 2 (T2), and tertile 3 (T3), respectively. Individuals in T2 seem to be the oldest, with 54.45% of them males. In addition, individuals in T1 had the lowest BMI, with higher education, more family income, and lowest smoking rate. The prevalence of hypertension, diabetes, prediabetes, coronary artery disease, and angina had no significant difference among T1, T2, and T3, while T3 occupied a highest proportion of stroke (26.25%) and T2 occupied a highest proportion of heart attack (50.11%).

**Table 2:** Characteristics of HF participants by tertiles of dietary inflammatory index (DII).

	**Tertiles of dietary inflammatory index**			
**Characteristic**	**T1, *n* = 460**	**T2, *n* = 461**	**T3, *n* = 461**	***p*-values**
	**(–3.884−0.570)**	**(–0.566–1.019)**	**(1.019–4.598)**	
**Age, years, Mean** **±*****SD***	68.23 ± 11.89	68.57 ± 12.24^b^^*^	66.67 ± 12.31	0.041
**Gender**, ***N*** **(%)**				
Male	322 (70.00%)	251 (54.45%)	205 (44.47%)	<0.001
Female	138 (30.00%)	210 (45.55%)	256 (55.53%)	
**BMI, kg/m**^**2**^, ***N*** **(%)**				
<20	10 (2.17%)	9 (2.10%)	15 (3.40%)	0.182
20–25	72 (15.65%)	63 (13.67%)	60 (13.02%)	
25–30	144 (31.31%)	123 (26.68%)	119 (25.810%)	
≥30	210 (45.65%)	239 (51.84%)	246 (53.36%)	
Mean ±*SD*	31.01 ± 7.21^a^^**^	32.58 ± 8.44	32.07 ± 8.41	0.013
Missing	24 (5.22%)	27 (5.86%)	21 (4.56%)	
Waist, cm, Mean ±*SD*	108.25 ± 16.23	110.25 ± 18.17	107.94 ± 16.60	0.119
**Race**, ***N*** **(%)**				
Mexican American	46 (10.00%)	38 (8.24%)	46 (9.98%)	<0.001
Other Hispanic	27 (5.87%)	30 (6.51%)	33 (7.16%)	
Non-Hispanic white	266 (57.83%)	256 (55.53%)	226 (49.02%)	
Non-Hispanic black	87 (18.91%)	121 (26.25%)	142 (30.80%)	
Other race or multi-racial	34 (7.38%)	16 (3.47%)	14 (3.04%)	
**Education**, ***N*** **(%)**				
<High school	141 (30.65%)	173 (37.53%)	216 (46.85%)	<0.001
High school	120 (26.09%)	114 (24.73%)	110 (23.86%)	
>High school	199 (43.26%)	174 (37.74%)	135 (29.28%)	
**Annual family income**, ***N*** **(%)**				
<20,000 USD	146 (31.74%)	184 (39.91%)	202 (43.82%)	0.001
≥20,000 USD	289 (62.83%)	255 (55.31%)	245 (53.15%)	
Missing	25 (5.43%)	22 (4.77%)	14 (3.04%)	
**Current smoking status**, ***N*** **(%)**				
Smoking	68 (14.78%)	79 (17.14%)	119 (25.81%)	<0.001
Non-smoking	232 (50.43%)	189 (41.00%)	167 (36.23%)	
Missing	160 (34.78%)	193 (41.87%)	175 (37.96%)	
**Hypertension**, ***N*** **(%)**	380 (82.61%)	387 (83.95%)	389 (84.38%)	0.280
**Diabetes**, ***N*** **(%)**	191 (41.52%)	202 (43.82%)	200 (43.38%)	0.959
**Prediabetes**, ***N*** **(%)**	32 (6.96%)	27 (5.86%)	23 (4.99%)	0.612
**Coronary artery disease**, ***N*** **(%)**	203 (44.13%)	189 (41.00%)	188 (40.78%)	0.343
**Angina**, ***N*** **(%)**	132 (28.70%)	124 (26.90%)	104 (22.56%)	0.157
**Heart attack**, ***N*** **(%)**	200 (43.48%)	231 (50.11%)	220 (47.72%)	0.016
**Stroke**, ***N*** **(%)**	93 (20.22%)	87 (18.87%)	121 (26.25%)	0.003

*Data are presented as N% (χ^2^-test) or Mean ± SD (independent t-test)*.

a, b *represents the post hoc between T1 and T2, T1 and T3, respectively*.

* *p < 0.05*,

** *p < 0.01*.

The physical and laboratory examinations in HF participants divided by tertiles are exhibited in Supplementary Table 2. Individuals in T1 had the lowest level of cholesterol compared with the T3 individuals (4.49 ± 1.16 vs. 4.75 ± 1.28 mmol/L, *p* < 0.01). In addition, the T1 group had the highest level of albumin (40.76 ± 3.52 g/L), hemoglobin (13.76 ± 1.65 g/dl), and hematocrit (40.83 ± 4.69%) among these groups, which possibly indicates that a lower DII score is correlated with a better cardiac function.

### Association Between HF and Dietary Inflammatory Index

The unadjusted and adjusted models are presented in Table 3. Model 1 was a crude model which shows that there is a statistically significant difference between increased odds of HF and higher DII score [OR (95% CI) = 1.140 (1.104–1.177), *p* < 0.001]. The odds rate in model 2 was 1.158 after it was adjusted for age, gender, and BMI, while model 3 was additionally adjusted for race, education, income, and smoking status. Although the association was slightly weakened, there was still a significant association between higher DII levels and the increase of HF (OR = 1.110, 95 CI% 1.060–1.163, *p* < 0.001) in model 3.

**Table 3:** Logistic regression analysis of DII on HF in participants with cardiac-cerebral vascular disease in NHANES (1999–2018).

	**OR**	**95% CI**	***p*-values**
**Model 1**			
**DII score**	1.140	1.104–1.177	<0.001
**Model 2**			
**DII score**	1.158	1.119–1.199	<0.001
**Age, years**	1.054	1.048–1.059	<0.001
**Gender**			
Male	Ref.	Ref.	<0.001
Female	0.606	0.538–0.682	
**BMI, kg/m**^**2**^	1.049	1.040–1.057	<0.001
**Model 3**			
**DII score**	1.110	1.060–1.163	<0.001
**Age, years**	1.048	1.041–1.056	<0.001
**Gender**			
Male	Ref.	Ref.	<0.001
Female	0.611	0.518–0.721	
**BMI, kg/m**^**2**^	1.051	1.040–1.062	<0.001
**Race**			
Mexican American	0.680	0.434–1.065	0.092
Other Hispanic	0.961	0.594–1.555	0.872
Non-Hispanic white	1.157	0.795–1.683	0.447
Non-Hispanic black	1.056	0.713–1.565	0.785
Other Race or Multi-racial	Ref.	Ref.	
**Education**			
<High school	1.276	1.056–1.542	0.012
High school	1.185	0.976–1.437	0.086
>High school	Ref.	Ref.	
**Annual family income**			
<20,000 USD	Ref.	Ref.	
≥20,000 USD	0.696	0.592–0.818	<0.001
**Current smoking status**			
Smoking	1.305	1.085–1.571	0.005
Non-smoking	Ref.	Ref.	

*The cardiac-cerebral vascular disease in the present study including: hypertension, diabetes, prediabetes, coronary artery disease, angina, heart attack, and stroke*.

### Random Forest Modeling of HF Using Laboratory Examinations and Dietary Data

**Figure 2:**
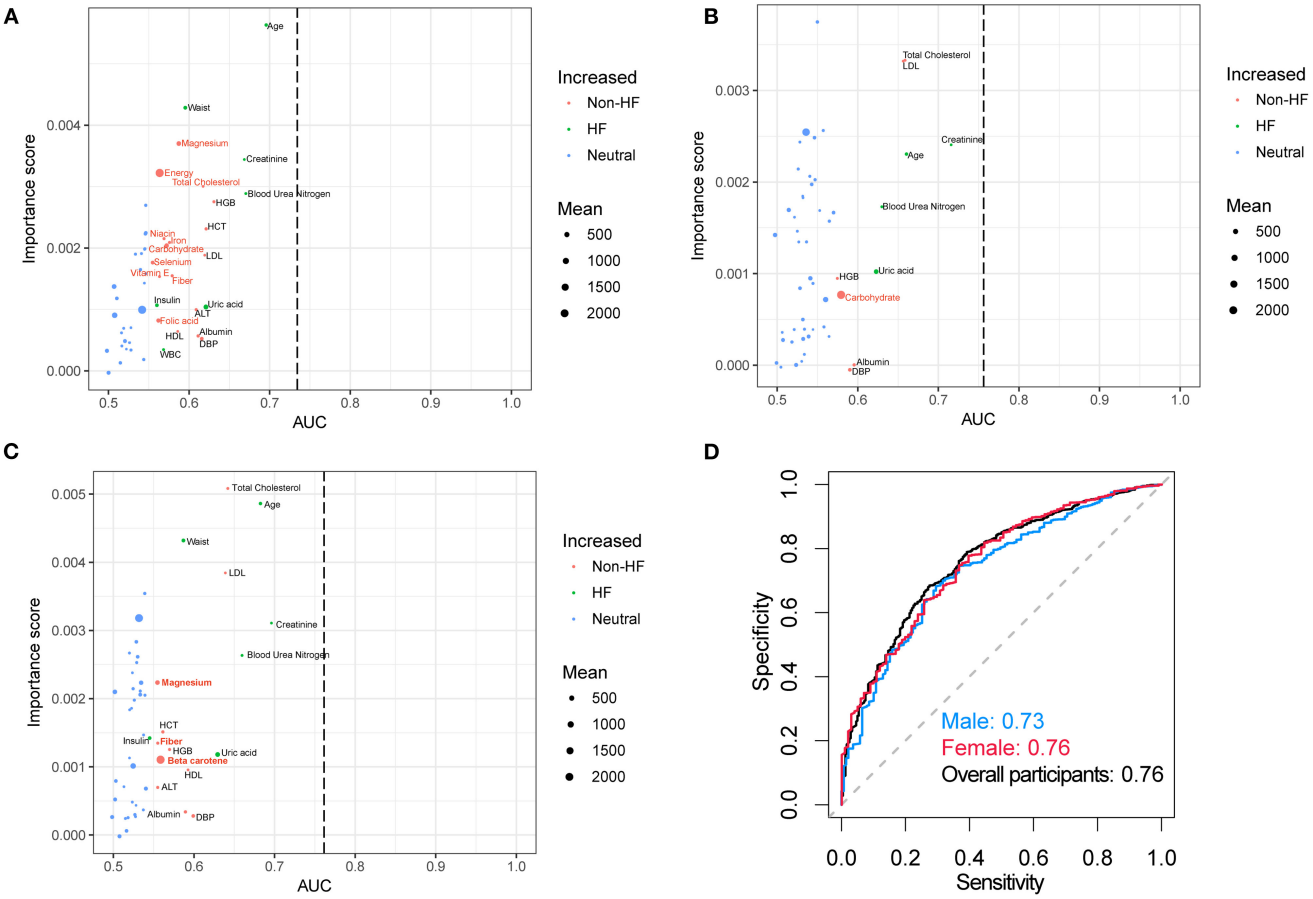
Random forest analysis and AUC curves. Random forest analysis in **(A)** male, **(B)** female, **(C)** overall participants and their corresponding **(D)** AUC curves. The green (HF group) and red (non-HF group) nodes which were significantly different between these two groups denoted the important score in the construction of the random forest model. The AUC curves for the random forest models in separating HF and non-HF groups using the dietary information and laboratory examinations. The red font indicated the dietary indices. AUC, area under the curve.

Random forest analysis discriminated HF participants from non-HF with an area under the curve (AUC) of 0.73, 0.76, and 0.76 using the laboratory examinations and demographic characteristics from male, female, and total individuals, respectively (Figure 2). The important dietary indices in constructing a random forest model in the male subgroup include magnesium, energy, total cholesterol, niacin, iron, carbohydrate, selenium, vitamin E, fiber, and folic acid. However, these important dietary indices disappeared in the model of the female subgroup except for carbohydrate, while magnesium, fiber, and beta carotene were important indices in all the participants. These results might suggest that dietary intervention is more essential for male. In addition, supplement with magnesium might be essential for HF.

## Discussion

In this study, we provide evidences that HF is closely associated with DII by using the NHANES 1999–2018 data, in which fiber, niacin, iron, selenium, vitamin E, β-carotene, and magnesium might be important dietary factors in HF. Nutrition is a factor that should not be ignored in the progress of HF. Malnutrition or cachexia is an independent risk factor which increases the mortality of HF (18). In fact, the failing heart is regarded as an engine out of fuels (19). Beyond the supply of raw materials for protein synthesis, nutrients also serve as energy sources for the heart. Therefore, supplement with sufficient nutrients is recommended at the end stage of HF, especially those patients who are accompanied with malnutrition (5). Nevertheless, the diet-related inflammation is an indispensable factor that needs to be a concern.

Previous studies have investigated the association between DII and metabolic syndrome or cardiovascular risk factors (20–22). Although HF is the terminal stage of various CVD, their pathological mechanism indeed might be different and fewer studies are concerned about DII and HF. Recently, growing evidences have indicated that the progress and prognosis of HF are influenced by gut microbiota and their metabolites (23). In addition, dietary intakes severely impact the composition of gut microbiota (24). For example, trimethylamine oxide, which is derived from a high choline diet by gut microbiota, is highly associated with HF (25). Focusing on the dietary consumption of HF patients might provide some valuable information.

In the present study, we observed that HF is more prevalent in individuals with a lower family income and lower education. However, a small sample size prospective study indicated that education levels were not associated with readmission or mortality rates (26). This discrepancy might be due to the difference of regions. From a multinational, prospective cohort study, the effect of low education on CVD is stronger in middle-income or low-income countries rather than high-income countries (27). Another interesting finding is that LDL, a traditional risk factor in CVDs, is reduced in HF individuals. The possible explanation is on the poor nutritional intake or appetite of HF patients (28). In addition, individuals in T1 of DII have a slightly increased level of serum albumin, hemoglobin, and hematocrit, which also supported that diet is essential in HF.

Furthermore, from the results of the random forest model, the dietary magnesium, fiber, and beta carotene might be nutritional elements that are beneficial to HF. Emerging evidences suggested that a low dietary magnesium intake is associated with an increased CVDs risk and the beneficial effects of magnesium supplements on the prevention of CVDs (29). However, the impact of magnesium might be more profound in males from our observation. In addition, a high fiber intake has been shown to be closely linked with gut microbiota and ameliorated cardiac function (30). Previous studies have demonstrated that lower concentrations of serum beta carotene may increase the risk of sudden cardiac death (31) and is associated with an increased risk of chronic HF (32). Diet intervention might be an easily modifiable method in the therapy of HF, while further investigation is needed to determine the underlying mechanism.

There are still some limitations that should be mentioned in the present study. First, the design is an observational study, which might lead to bias and lack of evidence of the cause and effect. Second, the diagnosis of HF is acquired by a questionnaire survey, and it is hard to assess the severity of HF. In addition, without the data of ejection fraction, the subtype of HF cannot be categorized.

In summary, we report an association between DII and HF, in which the dietary components should be a concern in the future.

## Data Availability Statement

The datasets presented in this study can be found in online repositories. The names of the repository/repositories and accession number(s) can be found at: NHANES 1999-2000 https://wwwn.cdc.gov/nchs/nhanes/continuousnhanes/default.aspx?BeginYear=1999; NHANES 2001-2002 https://wwwn.cdc.gov/nchs/nhanes/continuousnhanes/default.aspx?BeginYear=2001; NHANES 2003-2004 https://wwwn.cdc.gov/nchs/nhanes/continuousnhanes/default.aspx?BeginYear=2003; NHANES 2005-2006 https://wwwn.cdc.gov/nchs/nhanes/continuousnhanes/default.aspx?BeginYear=2005; NHANES 2007-2008 https://wwwn.cdc.gov/nchs/nhanes/continuousnhanes/default.aspx?BeginYear=2007; NHANES 2009-2010 https://wwwn.cdc.gov/nchs/nhanes/continuousnhanes/default.aspx?BeginYear=2009; NHANES 2011-2012 https://wwwn.cdc.gov/nchs/nhanes/continuousnhanes/default.aspx?BeginYear=2011; NHANES 2013-2014 https://wwwn.cdc.gov/nchs/nhanes/continuousnhanes/default.aspx?BeginYear=2013; NHANES 2015-2016 https://wwwn.cdc.gov/nchs/nhanes/continuousnhanes/default.aspx?BeginYear=2015; NHANES 2017-2018 https://wwwn.cdc.gov/nchs/nhanes/continuousnhanes/default.aspx?BeginYear=2017.

## Ethics Statement

Ethical review and approval was not required for the study on human participants in accordance with the local legislation and institutional requirements. Written informed consent for participation was not required for this study in accordance with the national legislation and the institutional requirements.

## Author Contributions

QX, WL, RT, ZL, and HL conceived the ideas and design of the study. ZL, HL, QD, and CS collected data from NEHANES. ZL, HL, WH, and WZ analyzed the data. ZL, HL, and RT drafted the manuscript. QX and WL revised the final version of the manuscript and supervised the study. All authors have read and approved the final version of the manuscript for publication.

## Conflict of Interest

The authors declare that the research was conducted in the absence of any commercial or financial relationships that could be construed as a potential conflict of interest.
