# Supplementary material for: Association Between Dietary Inflammatory Index and Heart Failure: Results From NHANES (1999–2018)
Source: Front Cardiovasc Med. 2021 Jul 6;8:702489. doi: 10.3389/fcvm.2021.702489 (PMC8292138; doi:10.3389/fcvm.2021.702489)
Supplement: Supplementary file 1 [file Table_1.docx]

Supplementary Table 1. Physical and laboratory examinations of participants, from NHANES 1999-2018

| Examination | HF  (N = 1382, 7.25%) | Non-HF  (N = 17685, 92.75%) | *P* values |
| --- | --- | --- | --- |
| Dietary inflammatory index | 0.239 ± 1.702 | -0.145 ± 1.704 | < 0.001 |
| WBC, 1000 cell/μL | 7.60 ± 2.66 | 7.38 ± 4.00 | 0.051 |
| Total Cholesterol, mmol/L | 4.61 ± 1.20 | 5.05 ± 1.14 | < 0.001 |
| Triglyceride, mmol/L | 1.77 ± 1.92 | 1.62 ± 1.34 | 0.055 |
| LDL-Cholesterol, mmol/L | 2.58 ± 0.99 | 2.91 ± 0.93 | < 0.001 |
| Fasting Glucose, mmol/L | 7.10 ± 2.79 | 6.72 ± 2.58 | 0.001 |
| Insulin, pmol/L | 121.17 ± 204.37 | 95.23 ± 125.81 | 0.003 |
| HDL-Cholesterol, mmol/L | 1.26 ± 0.41 | 1.36 ± 0.42 | < 0.001 |
| Albumin, g/L | 40.27 ± 3.66 | 41.63 ± 3.42 | < 0.001 |
| AST, IU/L | 26.50 ± 30.98 | 25.74 ± 16.81 | 0.382 |
| ALT, IU/L | 23.89 ± 41.04 | 25.06 ± 18.25 | 0.050 |
| Blood Urea Nitrogen, mmol/L | 7.43 ± 4.32 | 5.41 ± 2.45 | < 0.001 |
| Creatinine, mg/dL | 1.30 ± 0.96 | 0.97 ± 0.58 | < 0.001 |
| Globulin, g/L | 30.77 ± 5.62 | 30.01 ± 4.88 | < 0.001 |
| Sodium, mmol/L | 139.22 ± 3.01 | 139.23 ± 2.65 | 0.891 |
| Total bilirubin, μmol/L | 12.04 ± 5.60 | 11.27 ± 4.95 | < 0.001 |
| Uric acid, μmol/L | 386.22 ± 11.56 | 339.32 ± 88.29 | < 0.001 |
| Pulse rate, per 60 sec | 70.84 ± 12.46 | 72.59 ± 12.79 | < 0.001 |
| Systolic blood pressure, mmHg | 132.65 ± 23.48 | 132.62 ± 20.56 | 0.967 |
| Diastolic blood pressure, mmHg | 66.48 ± 16.20 | 71.10 ± 14.97 | < 0.001 |
| Hemoglobin, g/dL | 13.55 ± 1.72 | 13.98 ± 1.55 | < 0.001 |
| Hematocrit, % | 40.29 ± 4.90 | 41.35 ± 4.38 | < 0.001 |

Data are presented as Mean ± SD (independent t-test)
