# Supplementary material for: Association Between Dietary Inflammatory Index and Heart Failure: Results From NHANES (1999–2018)
Source: Front Cardiovasc Med. 2021 Jul 6;8:702489. doi: 10.3389/fcvm.2021.702489 (PMC8292138; doi:10.3389/fcvm.2021.702489)
Supplement: Supplementary file 2 [file Table_2.docx]

Supplementary Table 2. Examinations of HF participants by tertiles of dietary inflammatory index (DII)

|  | Tertiles of Dietary inflammatory index | | |  | |
| --- | --- | --- | --- | --- | --- |
| Examination | **T1**  **(-3.884 ~ -0.570)** | **T2**  **(-0.566 ~ 1.019)** | **T3**  **(1.019 ~ 4.598)** | | ***P* values** |
| Dietary inflammatory index | -1.653 ± 0.759^a***, b***^ | 0.218 ± 0.464 ^c***^ | 2.150 ± 0.819 | | < 0.001 |
| WBC, 1000 cell/μL | 7.59 ± 2.76 | 7.64 ± 2.88 | 7.57 ± 2.30 | | 0.933 |
| Total Cholesterol, mmol/L | 4.49 ± 1.16 ^b**^ | 4.58 ± 1.14 | 4.75 ± 1.28 | | 0.005 |
| Triglyceride, mmol/L | 1.72 ± 2.12 | 1.64 ± 0.98 | 1.94 ± 2.34 | | 0.237 |
| LDL-Cholesterol, mmol/L | 2.52 ± 0.92 | 2.55 ± 0.95 | 2.68 ± 1.08 | | 0.209 |
| Fasting Glucose, mmol/L | 7.03 ± 2.86 | 6.93 ± 1.94 | 7.35 ± 3.34 | | 0.284 |
| Insulin, pmol/L | 107.43 ± 143.44 | 127.33 ± 241.53 | 129.05 ± 217.75 | | 0.505 |
| HDL-Cholesterol, mmol/L | 1.30 ± 0.41 | 1.24 ± 0.41 | 1.26 ± 0.40 | | 0.322 |
| Albumin, g/L | 40.76 ± 3.52^a*, b***^ | 40.21 ± 3.58 | 39.86 ± 3.83 | | 0.001 |
| AST, IU/L | 27.40 ± 33.30 | 25.87 ± 12.94 | 26.26 ± 40.36 | | 0.753 |
| ALT, IU/L | 26.36 ± 67.45 | 22.60 ± 15.24 | 22.76 ± 18.26 | | 0.319 |
| Blood Urea Nitrogen, mmol/L | 7.36 ± 3.75 | 7.62 ± 4.24 | 7.29 ± 4.74 | | 0.506 |
| Creatinine, mg/dL | 1.25 ± 0.78 | 1.29 ± 0.83 | 1.37 ± 1.20 | | 0.217 |
| Globulin, g/L | 29.92 ± 5.37^a**, b***^ | 31.08 ± 5.56 | 31.29 ± 5.84 | | 0.001 |
| Sodium, mmol/L | 139.26 ± 2.94 | 139.05 ± 2.93 | 139.35 ± 3.16 | | 0.338 |
| Total bilirubin, μmol/L | 12.40 ± 5.81 | 12.01 ± 5.36 | 11.71 ± 5.63 | | 0.192 |
| Uric acid, μmol/L | 383.70 ±106.68 | 395.60 ± 110.25 | 379.07 ± 114.21 | | 0.075 |
| Pulse rate, per 60 sec | 69.90 ± 12.22^b**^ | 70.42 ± 12.30^c*^ | 72.22 ± 12.77 | | 0.014 |
| Systolic blood pressure, mmHg | 132.18 ± 22.99 | 131.82 ± 22.38 | 133.94 ± 25.01 | | 0.364 |
| Diastolic blood pressure, mmHg | 67.07 ± 15.89 | 65.26 ± 15.48 | 67.09 ± 17.15 | | 0.136 |
| Hemoglobin, g/dL | 13.76 ± 1.65^a**, b*^ | 13.42 ± 1.71 | 13.46 ± 1.77 | | 0.006 |
| Hematocrit, % | 40.83 ± 4.69^a**, b*^ | 39.87 ± 4.96 | 40.17 ± 5.01 | | 0.013 |

Data are presented as Mean ± SD (independent t-test)

^a, b, c^ represents the post hoc between T1 and T2, T1 and T3, T2 and T3. ^*^*p*<0.05, ***p*<0.01, ****p*<0.001
